# Working Memory Capacity as a Determinant of Proactive Interference and Auditory Distraction

**DOI:** 10.5334/joc.7

**Published:** 2018-01-10

**Authors:** Gerald Tehan, Madeleine Arber, Georgina Anne Tolan

**Affiliations:** 1 University of Southern Queensland, Ipswich, AU; 2 Australian Catholic University, Brisbane, AU

**Keywords:** Working memory, Speech perception, Attention

## Abstract

Individual differences in working memory capacity are related to performance on a range of elemental and higher order cognitive tasks. The current experiment tests the assumptions of two theoretical approaches to working memory capacity: working memory as executive attention and working memory as temporary binding. These approaches are examined using a short-term updating task where proactive interference is manipulated, such that old responses have to be suppressed in favour of new responses. A second source of distraction is introduced by way of irrelevant, to-be-ignored background speech that accompanies presentation of the list items. This speech reinforces either the to-be-remembered item on the current list, or the to-be-suppressed item. Working memory capacity was significantly related to overall level of correct performance on the short-term task, and to the degree of proactive interference experienced. However, there was no evidence for individual differences in the ability to suppress the interfering foil, nor in priming effects associated with the irrelevant speech. The results provided little support for the working memory capacity as executive attention perspective, some evidence for the binding perspective, but also evidence supporting the fact that some effects of distraction are not under voluntary control.

In 1974, Baddeley and Hitch suggested that working memory was a fundamental substrate of all cognitive activity. Subsequent research has confirmed that performance on working memory tasks shares substantial variance with both higher order and more elemental cognitive tasks. In recent times the emphasis has shifted to understanding individual differences in working memory capacity (WMC). Wilhelm, Hildebrandt and Oberauer (2013), for instance, have suggested that there are three broad approaches to understanding WMC: WMC as executive attention, WMC as Primary Memory and Secondary Memory, and WMC as binding. The current experiment is developed to compare the executive attention and binding approaches using a short-term cued recall task where resistance to proactive interference (PI) and resistance to distraction are manipulated.

The executive attention view of working memory is empirically based on working memory capacity differences across a range of distraction tasks such as dichotic listening tasks (Conway, Cowan, & Bunting, 2001), the antisaccade task (Unsworth, Redick, Spillers, & Brewer, 2012), and flanker tasks (Heitz & Engle, 2007). The attentional control account of WMC argues for a limited pool of attentional resources “whereby memory representations are maintained in a highly active state in the face of interference” (Kane & Engle, 2002, p. 638). This definition has been interpreted in two ways: capacity can be focused upon the target item such that distractors are not processed, or it can be used to inhibit or supress distractor activity. In subsequent research, Miyake et al. (2000) suggested that executive attention could divided into three separate but related factors, these being shifting, updating, and inhibition factors. Later research led Friedman and Miyake (2004) to subdivide the inhibition factor into a further three subsets involving pre-potent responses inhibition, as in the Stroop task; resistance to distraction from interference, as in the effects of irrelevant stimuli in tasks such as the flanker task; and in resistance to PI. Individual differences in WMC have been linked to all three forms, but the current paper is concerned primarily with resistance to PI and to distraction. Importantly, those who have high WMC show less PI in a range of memory tasks (Kane & Engle, 2002; Kliegl, Pastotter, & Bauml, 2015). The relationship between capacity and resistance to distraction is less clear with some studies showing a significant relationship between the two constructs (Unsworth, 2010) and others showing no significant relationship (Keye, Wilhelm, Oberauer, & van Ravenzwaaij, 2009).

According to the binding approach (Oberauer, Süß, Wilhelm, & Sander, 2007; Wilhelm et al., 2013), WMC reflects the ability for building, maintaining, and updating temporary bindings. For example, in memory for lists of words that involve multiple trials, items have to be bound to contextual elements such as “the current list” and/or “position within the list”. In some tasks, such as the n-back and running memory span tasks, these temporary bindings need to be continually updated. Thus, the formation of rapid temporary bindings “enables the system to construct and maintain new structures, such as random lists… the limited capacity of working memory arises from interference between bindings…” (Wilhelm et al., 2013, p. 4). Wilhelm et al., argued that updating tasks are the most appropriate measures of WMC because they involve all three binding functions: the formation and maintenance of new structures and the updating of those structures.

In the current study we examined attention and binding approaches using a short-term cued recall updating task. PI was manipulated within a single trial rather than across distinct trials as is typically the case in other PI paradigms. In this task (Tolan & Tehan, 2002) each trial consists of a small number of items, one (target) of which is a member of a taxonomic category. At test the category label is presented as a cue for recall of the relevant target. The list items are organised into a single block of four items on a small number of trials and more frequently into two blocks, the first containing four words and the second containing five words. In each case the instructions stressed that participants are to remember the items in the most recently presented block. PI is manipulated in this paradigm via the presentation in the to-be-forgotten first block of another member (foil) from the same taxonomic category as the target item in the second block. The effects of PI emerge in suppressed recall of the target item and in the enhanced recall of the to-be-forgotten foil, relative to performance on a no-interference condition where the target is the only instance of the taxonomic category in the list. The two-block trials are effectively an updating task, where participants remember some items for a brief period of time and then are asked to forget those items and remember the next set of items. From the binding perspective, participants form a temporary structure, the first block, and then when it is apparent that the task is a two-block trial, they forget the old structure and create a new temporary structure, the second block.

Two studies have explored individual differences in working memory capacity on the cued recall task (Friedman & Miyake, 2004; Unsworth, 2010), but neither involved all possible measures, and both studies employed a retention interval of eight seconds instead of the immediate test used in the current experiments. Friedman and Miyake (2004) compared performance on one-block trials to performance on no-interference two-block trials to measure interference, and Unsworth (2010) used performance on the one-block trials as a measure of no-interference and lure recall as the measure of interference. Both experiments reported that individual differences in working memory capacity, as measured by multiple complex span tasks, were related to resistance to proactive interference.

Irrelevant speech is the second factor that is manipulated in the current experiment. We will refer to the irrelevant speech effect in this paper, acknowledging that the irrelevant speech effect represents a specific instance of the more general irrelevant sound effect (Beaman & Jones, 1997). The irrelevant sound effect represents the detrimental effect that background sounds have on short-term, serial recall of visually presented items, even when instructions stress that the sounds should be ignored (Jones & Macken, 1993). Such auditory distraction is maximised if the auditory stream contains changing state sounds rather than a single, repeated, steady-state sound; an outcome that is known as the changing-state effect (Jones, Madden, & Miles, 1992). In the current experiment we have adopted changing-state speech as the auditory distractor to involve the language system. We are interested in the effects that semantic and phonological features of the auditory distractors have on short-term recall, rather than the sensory features of the distractors.

Both attention and binding explanations of the irrelevant speech effect have been proposed. The attention control account assumes that changing state auditory input captures attention, and diverts resources from the primary memory task. Psychophysiological evidence using event-related potentials have supported such a view (Bell, Dentale, Buchner & Mayr, 2010), as has the demonstration that participants can habituate to the auditory stream (Röer, Bell, & Buchner, 2014). One important aspect of the attention account is that content similarity between the two streams should have little impact upon performance as it is attention capture that is the locus of the effect. Secondly, the approach does not automatically lead to specific predictions concerning the frequency with which different types of errors should be made. If high capacity participants are more able to focus attention on the target or alternatively, to suppress distractor activity, one might expect that there would be better target recall. Our reading of the theory is that it does not make any predictions concerning the frequency of different types of errors.

The binding account of irrelevant speech effect assumes that temporary bindings between contextual elements and items are disrupted by irrelevant speech. Much of the literature associated with the irrelevant speech effects involves item to order bindings, but Bell, Röer, and Buchner (2013) have shown that item to background colour bindings are just as susceptible to irrelevant speech as item to order bindings. Tolan and Tehan (2002), using the cued recall task described earlier, showed that PI effects, both in terms of diminished target recall and increases in foil recall, were more prevalent under irrelevant speech conditions than quiet conditions. Thus, irrelevant speech disrupted the item to block-2 context bindings such that on a number of trials, the foil in block-1 was incorrectly recalled.

Tolan and Tehan (2002) explored another aspect of irrelevant speech. They manipulated the content of the irrelevant stream to modulate the degree of PI. They were able to demonstrate that the auditory distractors could essentially prime either the target item or the interfering foil in the cued recall paradigm. When the speech supported the target item, performance was immune to PI, target recall was excellent and there were few block-1 intrusions. However, when the auditory items supported the foil, target recall was depressed and the foil was frequently recalled instead of the target. Their explanation for these effects involved the interaction of phonological and semantic codes in semantic memory (Tehan, Humphreys, Tolan & Pitcher, 2004; Tolan & Tehan, 2002). An emergent prediction from their explanation is that these between-stream priming effects are obligatory and are not under attentional control. This prediction has yet to be tested.

Individual differences in the strength of the irrelevant speech effect have been the focus of a considerable amount of research (see Sörqvist and Rönnberg, 2014, for a review). This literature confirms that those who have high capacity are less susceptible to irrelevant speech on many tasks, especially those that involve attention capture. Importantly, Beaman (2004) found that high WMC participants were less likely to show positional intrusion errors from previous lists in an immediate serial recall task than low WMC participants. This suggests that WMC is related to the frequency of PI errors as well as target recall.

However, there is one key feature of the irrelevant speech effect that is not related to WMC. Those who have high working memory capacities are just as susceptible to the changing state irrelevant speech effect as those with low working memory capacities (Beaman, 2004; Sörqvist, Marsh, & Nöstl, 2013). This suggests that some aspects of the irrelevant speech affect are not under attentional control.

In the current experiment, individual differences in WMC on the Tolan and Tehan (2002) task are examined under conditions where the irrelevant speech primes either the target item or the to-be-forgotten foil. From the executive attention perspective, those who have high WMC should be resistant to PI either through focusing purely upon the target recall or alternatively, they are better at suppressing distractors. High capacity participants should be immune to the effects of PI. Likewise, if auditory distractors are suppressed or not processed, the irrelevant speech priming effects should not be evident. From the binding perspective, those with high working memory capacity should be better on the updating task which would be reflected in better target recall, fewer omissions, and perhaps fewer block-1 intrusions (Beaman, 2004). However, the approach is silent about priming effects and what would happen if binding breaks down and the foil is recalled. The Tolan and Tehan (2002) account makes the prediction that priming effects are an emergent feature of the cognitive architecture that supports short-term recall and as such are obligatory and not susceptible to individual differences in either attentional control or binding.

## Methods

### Participants

Participants were 80 undergraduate psychology students at the Australian Catholic University, who participated for partial course credit.

### Materials

**Figure 1:**
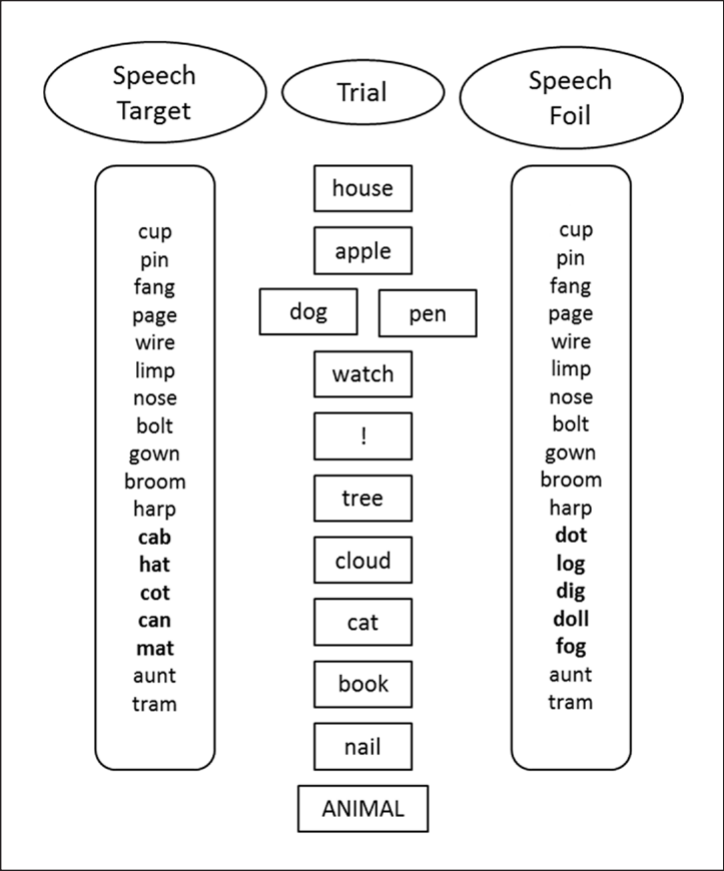
Structure of a standard trial with to-be-remembered items in the centre column, and irrelevant speech conditions on the outsice columns.

The materials and procedure used by Tolan and Tehan (2002) were utilised and are fully described in that paper. The cued recall component of the experiment consisted of 12 one-block trials and 40 two-block trials that were randomly ordered. The structure of the critical two block trials and the corresponding auditory distractors is presented in Figure 1. The critical two-block trials consisted of four words in the first block and five words in the second block. The two blocks were separated by an exclamation point (!) and the category cue presented in capital letters followed the end of the second block prompting recall. On all trials a single target item (in Figure 1 *cat*) was presented among fillers in the most recent block. Proactive interference was manipulated on 20 trials by including a second item (foil – *dog*) from the same category as the target in the first block. An example of an interference trial would be: *house, apple, dog, watch ! tree, cloud, cat, book, nail ANIMAL*. The other 20 trials were no-interference trials, in which the foil was replaced with a filler (*pen*). There are three measures of performance on this task: correct recall of the target, omissions where the target is not recalled (and nor is the foil), and false recall of the foil that appeared in the first block. Data from our lab indicates that twenty minute test-retest reliability estimates for the three measures are .65, .65 and .50 for target, omissions and lures, respectively.

All the cued recall trials were studied in the presence of background speech. The irrelevant speech consisted of a string of 18 words, five of which shared phonological features with either the target or the foil. These five items were presented in positions 12 to 16 in the irrelevant stream. Two of the five key irrelevant speech items shared the same stem as the foil or target, two shared the same rhyme ending and one shared the same consonants. An example of the critical irrelevant speech items supporting the target word, *cat, were **ca**b, h**at, c**o**t, ca**n*, and *m**at***. The irrelevant speech supported the foil on 10 of the interference trials and on 10 of the no-interference trials (although the foil was not actually present for these no-interference trials). Similarly, the speech supported the target on 10 of the interference trials and 10 of the no-interference trials.

The individual difference manipulation consisted of a modified operation span task in which words and math problems [e.g., (3 × 4) – 1 = 10] were presented in pairs. Participants read the word (and attempt to remember it) and then processed the mathematics problem. After all pairs were presented participants attempted to recall the words in the order in which they were presented. The current procedure deviated from the standard span methodology in that each trial consisted of four word/maths problems (fixed list length), and there were eight trials in total. Unpublished research from our laboratory has indicated that eight fixed-length trials of four words produces the same level of reliability as a 20-trial, span procedure starting with four trials at two words and finishing with four trials of six words. The twenty-minute test-retest reliability estimates were .79 and .87 for the maths and memory component respectively.

### Procedure

All participants were presented with the cued recall task first. Participants were instructed on the structure of the test and were told that they were to remember the most recent block of items. On the two-block trials a block-separator (!) was used to indicate that the trial was a two-block trial and that the participants should forget the first block and concentrate on the second block as it was this block that contained the target item. The items on each trial were presented at a rate of one item per second in the centre of a computer screen. The category cue was presented immediately after the final item for two seconds and the participant had 12 seconds to respond before the next trial began. During this period, participants could change their response if they chose to, but such events were extremely rare. At the start of the trial, the irrelevant speech commenced and finished with the presentation of the category cue. Participants were told to ignore the auditory material as best they could.

In the operation span task, the words/math problem on each trial were visually presented at a rate of three seconds per work/maths problem pair. Participants read each word aloud and then processed the maths problem. Following presentation of all pairs, a row of question marks appeared and the participant attempted serial recall.

## Results

In scoring the operation span task, two measures were generated. The first involved scoring an item as correct only if it was recalled in the correct serial position (order scoring). The second involved a free recall criterion, in which an item was scored as correct if it was recalled at all, irrespective of which position it was recalled in (item scoring). Internal consistency estimates were higher for the item scoring, α = .75, than for order scoring, α = .65, and consequently later analyses are based upon the item score. Table 1 presents the correlations between working memory measures and total scores on the cued recall task, including correct target recall, omissions, and recall of the block-1 foils. The correlations of WMC with the four individual types of cued recall trials showed identical outcomes to the total scores. Those who performed well on the working memory task also performed well on the cued recall of the target and made fewer omission errors. However, working memory scores were not significantly related to foil intrusions.

**Table 1:** Descriptive statics for and correlations between working memory measures and performance on the cued recall task.

	Mean	SD (Range)	WM Ord	WM Item	Cued Total	Omission Total

WMOrder	10.79	4.42 (3–25)				
WMItem	15.25	4.41 (6–26)	**.79**			
Cued Total	21.11	7.19 (5–35)	**.49**	**.53**		
Omissions Total	12.70	6.43 (0–34)	**–.38**	**–.47**	**–.86**	
Foil Total	4.16	2.08 (0–10)	–.11	–.04	**–.32**	–.12

*Note:* WM Ord = operation span order scoring, WMItem = operation span item scoring, Cued Total = total targets recalled, Omissions Total = neither target nor foil recalled, Foil Total = Total block-1 foils recalled instead of target. Correlations in bold font are significant at p = .05.

Two complementary sets of analyses were undertaken to assess the role of working memory capacity. In the first instance, a frequently used extreme groups design was employed to directly compare those with high working memory capacity with those low working memory capacity. In this design, the upper and lower quartiles of a distribution of WM span scores are categorized as high and low span, respectively. While this design has a number of strengths, it also has weaknesses (see Conway et al., 2005, for a review), not least that data from half the participants are not used, and continuous capacity variable is turned into a categorical variable with the consequent loss of specificity. To overcome these potential problems, our complementary ANCOVA used data from all participants and working memory capacity was used as a continuous variable. In this analysis, the variance due to working memory capacity was first extracted with the remaining variance then being analysed. Under these conditions, effects that are influenced by WMC should be severely attenuated as reflected in smaller F values and, more importantly, in diminished effect sizes.

Performance on the cued recall task is summarised in Table 2. The data replicate Tolan and Tehan’s (2002) finding that target recall was better in the no-interference condition than in the interference condition. Irrelevant speech also had an impact in that when the irrelevant speech supported the foil, target recall was depressed and false recall of the foil was enhanced relative to the conditions where the speech supported the target item.

**Table 2:** Mean performance on the cued recall task as a function of working memory capacity (WMC), proactive interference and irrelevant speech priming.

WMC	Interference	Speech Priming	
		
		Target	Foil

**Target Recall**			

High WMC	Interference	6.71 (1.93)	5.24 (2.30)
	No Interference	8.24 (1.09)	5.76 (2.14)
Low WMC	Interference	5.35 (1.70)	2.39 (2.10)
	No Interference	5.30 (2.40)	3.09 (2.11)
**Omission Errors**			

High WMC	Interference	2.10 (1.51)	1.62 (1.32)
	No Interference	1.76 (1.09)	3.10 (1.76)
Low WMC	Interference	3.35 (1.80)	3.91 (2.04)
	No Interference	4.35 (2.06)	4.70 (2.18)
**Foil Recall**			

High WMC	Interference	1.14 (0.96)	3.00 (1.61)
Low WMC	Interference	0.83 (0.98)	3.52 (1.92)

The cued recall target and omission data were first analysed by means of 2 (WMC) × 2 (PI) × 2 (irrelevant speech) mixed design ANOVAs. Likewise, the foil data were analysed via a 2 (WMC) × 2 (irrelevant speech) ANOVA that compared the two conditions that contained interfering foils. In both instances planned comparisons involved direct assessment of proactive interference and speech priming effects for both high and low working memory capacity groups.

The outcomes of the overall ANOVA presented in Table 3 and the outcomes of the planned comparisons are presented in Table 4. For target recall, high working memory capacity participants recalled more targets than the low capacity group, target recall was better when the speech supported the target than the foil, and target recall was better in the no interference condition than the interference condition. There was a group by interference interaction, but not a group by speech priming interaction. As Table 4 indicates, there were strong interference effects for the high working memory capacity group but not for the low working memory capacity group. Both groups showed speech priming effects that were of a similar magnitude. The ANCOVA (see Table 3) indicated that working memory capacity accounted for all interference effects, but there were still strong speech priming effects.

**Table 3:** ANOVA outcomes for target recall, omission errors and foil intrusions as a function of working memory capacity (WMC), proactive interference, and speech priming.

	High V Low WMC			WMC as Covariate		

	F (1,41)	p	η_p_^2^	F (1,78)	p	η_p_^2^

**Target**						

*WMC*	26.30	<.001	.39	30.86	<.001	.28
*PI*	12.18	.001	.23	.12	.734	.01
*Speech Priming*	81.27	<.001	.67	28.97	<.001	.27
WMC * PI	4.94	.032	.11	1.67	.200	.02
WMC * Priming	1.47	.232	.04	3.58	.062	.04
PI * Priming	0.03	.862	.01	1.12	.296	.01
WMC * PI * Priming	3.01	.090	.07	1.47	.229	.02
**Omissions**						

*WMC*	18.11	<.001	.31	22.16	<.001	.22
*PI*	16.10	<.001	.28	.94	.334	.02
*Speech Priming*	11.25	.002	.22	2.39	.126	.03
WMC * PI	0.84	.365	.02	.01	.916	.00
WMC * Priming	0.01	.938	.00	.07	.790	.01
PI * Priming	2.59	.115	.06	.13	.718	.00
WMC * PI * Priming	4.76	.036	.10	1.57	.215	.02
**Foil**						

*WMC*	0.00	.992	.00	.10	.750	.00
*Speech Priming*	56.25	<.001	.58	22.30	<.001	.22
WMC * Priming	1.86	.180	.04	3.47	.066	.04

**Table 4:** Comparisons of interference and speech priming effects for High and Low WMC groups on target recall, omissions and foil intrusions.

	Proactive Interference			Speech Priming		
	
	*t*	*p*	*Cohen’s d*	*t*	*p*	*Cohen’s d*

**Target**						

High WMC	4.13	.001	.74	6.51	<.001	1.20
Low WMC	0.88	.389	.13	6.46	<.001	1.32
**Omissions**						

High WMC	1.33	.198	.34	2.63	.016	.30
Low WMC	5.47	<.001	82	2.17	.041	.21
**Foil**						

High WMC				4.47	<.001	1.19
Low WMC				6.12	<.001	1.80

The results for the omission data were largely the mirror of the target outcomes. Low capacity participants made more omissions than those in the high capacity group, there were more omissions in the interference condition than the no interference condition, and there were more omissions made when the speech supported the foil than when it supported the target. The planned comparisons indicated that interference effects were not present for the high capacity group, but were for the low capacity group. Again speech priming effects were present in both groups and of a similar magnitude. The ANCOVA indicated that working memory capacity accounted for all the omission effects. That is, the differences in the proportion of omissions were fully explained by individual differences in working memory capacity.

In contrast to the other measures, high and low capacity groups did not differ in the number of foil intrusions. The intrusions were more frequent when the speech primed the foil than when they primed the target, and, as Table 4 indicates, this was true of both groups. The ANCOVA again confirmed that strong speech priming effects were still present after the effects of working memory capacity had been partialled out.

## Discussion

The cued recall paradigm permitted the examination of individual differences in WMC on two potential sources of interference within the one trial, PI and irrelevant speech. The outcomes of the experiment converge on five findings. Firstly, target recall was related to WMC. Those who performed well on the working memory component of the task also recalled the target item on the cued recall task more frequently than the low WMC participants. Secondly, and paradoxically, the superior ability of the high WMC group to recall the target, meant that they were more susceptible to PI than the low WMC group. To a large extent the low WMC group showed that they were not able to resolve competition between the target, auditory distractors and other list items resulting in high levels of omission errors. Thirdly, the ANCOVA analyses confirmed the role of WMC in PI, to the extent that PI effects in target recall were eliminated, as were the frequency of omissions across all experimental conditions, when variance in WMC had been partialled out. Fourthly, omissions and foil intrusions were not correlated and differences in capacity had no impact upon recall of the interfering foil. Lastly, the irrelevant speech priming effects were present in the data for both target and foil recall that were independent of WMC. In short, individual differences in WMC were reflected in the ability to remember the target item, but were not related to either foil recall or irrelevant speech priming effects.

From a theoretical perspective, the executive functioning account of WMC argues that high capacity participants are better able to focus on the target item by either supressing distractors or not processing these distractors (Kane & Engle, 2002). Irrelevant speech effects were present in the data and high WMC participants were better able to focus on the target item, an outcome that is consistent with the executive attention approach. There was less supporting evidence for the suppression of distractors. Irrelevant speech priming effects should not have emerged if the irrelevant stream had been suppressed. Likewise, there should have been correlations between WMC and foil intrusions if there were differences in ability to suppress the foil. However, priming effects were evident and WMC was independent of foil recall, a finding that is at odds with Unsworth’s (2010) findings of a correlation between foil recall in the cued recall task an WMC, and Beaman’s (2004) finding of a similar correlation involving the frequency of prior-list intrusions. One possible explanation for this discrepancy involves the content of the irrelevant speech. In the Unsworth experiment irrelevant speech was not manipulated, and in Beaman’s study, the content of the irrelevant speech was not manipulated. Manipulating the content of the irrelevant speech enhances the production of the block-1 foils to much higher levels than normal (Tolan & Tehan, 2002), suggesting that other processes are involved in the production of the foil. The current outcomes do confirm recent suggestions that there are boundary conditions to attentional control and susceptibility to distraction (Meier & Kane, 2015).

The results are more consistent with the binding account of WMC (Oberauer et al., 2007). According to this account high capacity participants are better at forming and maintaining new temporary structures (Wilhem et al., 2013). In the current task, these new structures would involve information about the items in the most recent block. In this task, not only do participants have to create and update new structures across trials, they have to update and create new structures within a trial. Given the task instructions, the binding operations in the PI task revolve around updating what the current target(s) are. As such target recall is the locus of where individual difference effects should be observed and it is where they were found.

Our interpretation of this approach is that it is silent about what happens when such binding breaks down. Our data provides some insight into the processes involved. WMC completely accounted for the omission data when there are breakdowns in the maintenance of the current structure (see Marsh, Sörqvist, Hodgetts, Beaman, & Jones, 2015, for other instances of WMC effects on target recall and current structure errors). However, WMC was not related to the degree of cross talk between old and new structures as reflected in foil intrusions, an outcome that is at odds with the notion that it is competition among bindings that is the locus of individual differences. The binding account is currently silent about sources of distraction that are not integral to the formation and maintenance of new temporary structures. It is not apparent how the binding approach would explain the irrelevant speech priming effects in particular, or more general changing state irrelevant speech effects (Beaman, 2004). How irrelevant speech interferes with the maintenance of temporary structures remains to be elucidated, but Bell et al. (2013) suggest that the irrelevant speech diverts attention away from the binding of an item and its context, and the maintenance of such bindings.

While we think the data are more consistent with the binding account, it is not certain that the two accounts are mutually exclusive. Many of the tasks that have explored distraction effects from the executive attention account do not rely upon the formation of item-to context bindings for discriminative purposes, but rather in the speed of item processing. In the cued-recall task the formation, updating and maintenance of temporal bindings is an essential component of the task. It is quite possible that differences in attentional control could result in stronger initial bindings, faster or more complete updating, better maintenance of such bindings, or all three processes. Thus, in this task, attentional control is not about item activation, but context to item bindings as suggested by Bell et al. (2013).

The fact that irrelevant speech priming effects were unrelated to and unaffected by individual differences in WMC, strongly indicates that these effects are largely not subject to attentional control. We have argued previously (Tolan & Tehan, 2000; Tehan et al., 2004) that these priming effects are an emergent property of the interaction of phonological and semantic features of items in a composite semantic memory where features of different items in the episode contribute to the construction of any given memory, whether those items are central to the memory task or are peripheral to it. As such the current priming effects (and changing state effects) are obligatory. The assertion that phonemic and semantic codes interact is not unique to the specific explanation here. Similar interactions in semantic memory have been proposed to account for speech production errors (e.g., Dell & Reich, 1981), priming in sentence recall (e.g., Schweppe, Rummer, Bormann, & Martin, 2011), and specific patterns of neuropsychological deficits (e.g., Martin, Lesch, & Bartha, 1999).

We have argued that our results address the relationship between working memory capacity, proactive interference and auditory distraction. While there is a considerable history of studies where performance on the operation span task has been the basis for inferences regarding the relationship between working memory capacity and other cognitive abilities, it is clear that no single working memory task, including the operation span task, is a pure measure of working memory capacity. Relying upon a single measure of verbal working memory, rather than a latent measure derived from multiple measures of working memory, represents a limitation of the current research. As such, the role of working memory capacity in proactive interference and auditory distraction in the cued recall task awaits confirmation with a more diverse range of complex span tasks from which a latent measure of working memory capacity can be obtained (Foster, Shipstead, Harrison, Hicks, Redick, & Engle, 2015).

The current experiment tested the widely held view that a defining function of working memory is the ability to protect cognitive activity from distraction. We show that WMC was related to the ability to form and maintain temporary episodic bindings that permitted the participants to isolate one episodic event. We also show that some forms of distraction are not under attentional control but are an emergent property of the cognitive architectures that support the recall process.

## Data Accessibility Statement

Data for this experiment is available for inspection at https://osf.io/tdyxp/.
